# Quantum mechanical calculation of nanomaterial-ligand interaction energies by molecular fractionation with conjugated caps method

**DOI:** 10.1038/srep44645

**Published:** 2017-03-16

**Authors:** Dawei Zhang

**Affiliations:** 1 School of Physics and Engineering, Henan University of Science and Technology, Luoyang 471023, P. R. China

## Abstract

Molecular fractionation with conjugate caps (MFCC) method is introduced for the efficient estimation of quantum mechanical (QM) interaction energies between nanomaterial (carbon nanotube, fullerene, and graphene surface) and ligand (charged and neutral). In the calculations, nanomaterials are partitioned into small fragments and conjugated caps that are properly capped, and the interaction energies can be obtained through the summation of QM calculations of the fragments from which the contribution of the conjugated caps is removed. All the calculations were performed by density functional theory (DFT) and dispersion contributions for the attractive interactions were investigated by dispersion corrected DFT method. The predicted interaction energies by MFCC at each computational level are found to give excellent agreement with full system (FS) calculations with the mean energy deviation just a fractional kcal/mol. The accurate determination of nanomaterial-ligand interaction energies by MFCC suggests that it is an effective method for performing QM calculations on nanomaterial-ligand systems.

The growing interest to study the electronic structures of large and complex systems such as protein, DNA, RNA, nanomaterials and other polymers in an efficient manner leads to the development of many computational schemes, which can cope with the limitation of computational resources. Linear scaling methods have been of particular interest because they allow large systems to be treated by quantum mechanics (QM). In particular, the use of quantum fragmentation is a very attractive tool to deal with large macromolecular systems^1^,^2^,^3^,^4^,^5^.

In quantum fragment-based approach, a macromolecule is divided into small fragments and simple computations of individual fragments are combined to achieve properties of a whole molecule. The earlier approach of quantum fragmentation for large systems is divide-and-conquer (DAC) method^6^,^7^,^8^, which was introduced by Yang *et al*. in 1991. In this method, an entire system is divided into subsystems and a buffer region is introduced into each subsystem to account for the interaction between the adjacent subsystems. Later, fragment molecular orbital (FMO) approach^9^,^10^,^11^,^12^,^13^ was proposed for *ab initio* calculations of large biomolecules. In this approach, a biomolecule is divided into a collection of small fragments and MO calculations are performed for each fragment and fragment pairs. One advantage of FMO method is that it can be performed with a number of QM techniques, thus having flexibility in terms of choice of methods for each system. However, FMO method may meet difficulties in the treatment of transition metal bearing systems and elaborate manual efforts are required.

Theoretical chemists have also come up with many other approaches to achieve QM calculations for large molecules. One famous example for calculating large molecules is fragment-based hybrid quantum mechanics/molecular mechanics (QM/MM) approach^14^,^15^,^16^,^17^ which uses force fields for the larger part of the system and performs more expensive QM calculations for the smaller remaining part. Later, the QM/MM technique within ONIOM framework was developed to carry out large scale modeling in a cost-effective manner^18^,^19^,^20^,^21^. However, the subsystem that can be treated by QM is limited in most currently available applications.

In recent years, there has been a surge of interests in developing other categories of linear scaling methods to treat complex systems quantum mechanically. Several non-fragmentation-based linear-scaling DFT methods have been developed to overcome the computational scaling of conventional DFT methods and at the same time provide the accuracy of conventional first-principle methods at a fraction of the computational cost for large systems. Examples of widely-used codes include CP2K^22^, ONETEP^23^, SIESTA^24^ and OpenMX^25^. These approaches have the added advantage of being able to compute forces and perform geometry optimizations and *ab initio* dynamics. As an alternative, MFCC based approach for the QM calculations of biomaterials was developed by Zhang *et al*. in 2003^26^. The basic idea of MFCC method is dividing a whole system into fragments and conjugated caps. The properties of the whole molecule can be obtained from the summation of standard QM calculations of the fragments from which the contribution of the conjugated caps is removed. The computational effort in the MFCC approach scales linearly with the molecule size, making it practical to deal with realistic large macromolecular systems. This method has been used for the calculation of interaction energies of protein with water^27^, drugs^28^, and ligands^29^. As well as, this method has been successfully applied to compute energies of charged molecules and large sized molecules such as long oligomers of *trans*-polyacetylene with up to 400 atoms at the MP2 and HF levels^30^,^31^. In a recent study, *ab-initio* calculation of large protein-ligand systems with 3680, 1798 and 1060 atoms which are beyond the reach of traditional computational methods has been made possible by using the MFCC approach^32^. Initially, MFCC approach was employed only to study biomolecules, but later on MFCC based attempt to study nanomaterials was reported by Li *et al*. in 2005 in which BN nanotubes were optimized using both MP2/6-31G* and HF/3-21G levels and the predicted total energy of unit BN and infinite BN nanotubes by MFCC were found to be almost equal to the extrapolated values from conventional MP2 and HF calculations on smaller tubes^30^.

Studying nanomaterials by QM fragmentation is highly beneficial from the point of view of accurate analyzing and understanding the chemistry of these large systems. Several other QM fragmentation methods have been introduced in the past for the calculation of properties of these large molecules. In 2006, molecular tailoring approach (MTA) was developed by Gadre *et al*.^33^,^34^ to investigate the structures, energetics and reactivity of boric acid nanotubes. Later on, MTA approach for large conjugated systems was reported for one-dimensional and two-dimensional π conjugated molecules by introducing a systematic algorithm for tailoring such systems^35^. The geometry optimization of these systems within MTA framework was attempted and the generated geometries were found to be in good agreement with their actual counterparts. In 2007, another new fragmentation approach based on DAC strategy was developed by Nakai research group^36^. They performed DFT calculations of polyene chains C_n_H_n+2_ (n = 30–240) using a new DAC method at HF, B3LYP and BLYP levels and found out absolute energy differences between conventional and the new DAC method are comparatively small. In 2009, FMO method was applied to study geometry optimization of a silicon nanowire Si_224_H_162_ at several levels of theory^37^. During the study, the nanowire was divided into 6 fragments of similar size and optimized with a good agreement to experimental results. When reducing the fragment size by dividing the nanowire into 12 fragments, the energy was not well reproduced. However, most of these QM fragmentation methods were applied only for linear molecules, indicating the necessity of the development of an appropriate method which can be employed to study various forms of nanomaterials.

Current methods for computation of nanomaterial ligand interaction energies may be limited and complicated. In a recent study, Car-Parrinello molecular dynamics (CPMD) method was used to calculate interaction energies of isolated and periodic boron nitride nanotubes (BNNTs) with and without water respectively^38^. When using a force field with the existing Lennard-Jones parameters, the QM interaction energies of water with BNNT can not be reproduced. It was clear that both boron and nitrogen parameters need to be adjusted to obtain a good agreement with the QM energies. Modification and fitting of parameters is complicated and a time consuming process, hence development of a simple and efficient way to calculate nanomaterial-ligand interaction energies is becoming more and more important. Here we present MFCC as an alternative approach for the efficient computation of the interaction energies of tube (carbon nanotube), sphere (fullerene) and surface (graphene) type nanomaterials with both neutral and charged ligands. The main purpose of this study is to prove the applicability and efficiency of MFCC approach to study the intermolecular interactions of different types of nanomaterials by partioning them into smaller fragments. This paper is organized as follows. Section II gives details of MFCC approach and fragmentation procedures of carbon nanotube (CNT), fullerene and graphene surface. In section III, we test the performance of MFCC and make comparison with full system (FS) calculations. In this section, we will test the accuracy and applicability of the MFCC approach within different computational levels such as B3LYP/6-31G* and B97D/6-311G*. The purpose of these calculations is to verify the accuracy of MFCC approach to provide information on different computational levels to account for the nanomaterial-ligand interactions. Finally, a summary is given in section IV.

## Computational Methods

Below we briefly outline the overall procedure for constructing subsystems for a given nanomaterial-ligand system. The theoretical background of MFCC approach for the interaction energy calculations of nanomaterials is explained using the CNT-ligand, fullerene-ligand and graphene-ligand systems respectively.

### CNT-ligand system

**Figure 1:**
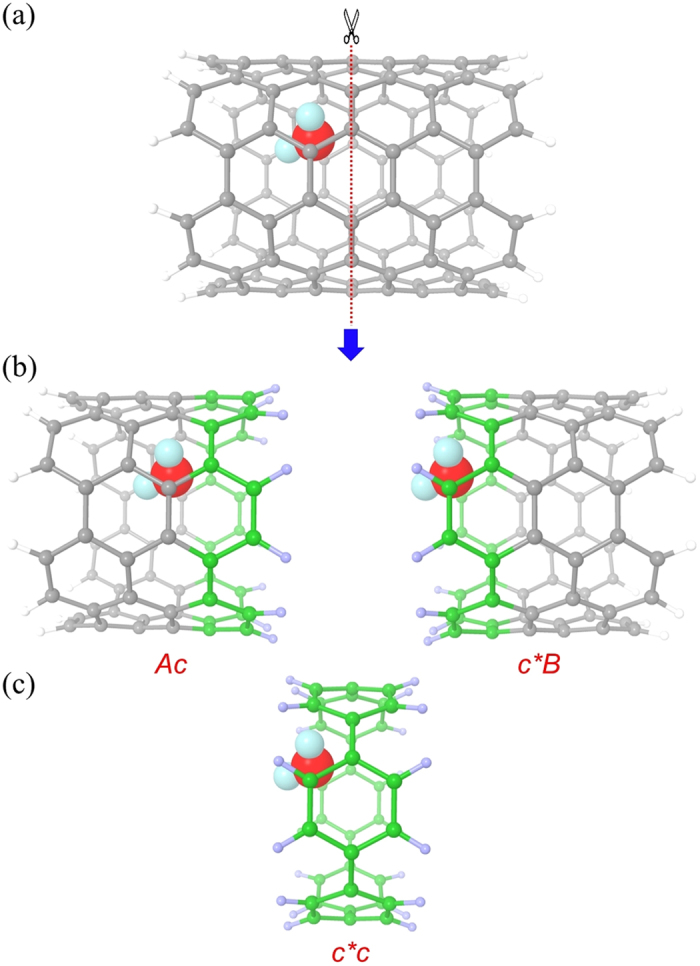
The MFCC scheme of CNT in which (**a**) a CNT is cut along the red dashed line, (**b**) a pair of concap (color green) is inserted to cap the fragments and (**c**) the concap is fused to form molecular concap species.

CNT has an infinite long structure comparable to large macromolecules, which gives an ideal example to test the accuracy of MFCC method. During the fragmentation process, we selected a part of long (6, 6) armchair CNT with diameter 8.0 Å and length 9.8 Å without losing the generality. Figure 1 illustrates the MFCC scheme in which the CNT is cut from the middle to produce A/B components, a pair of caps c/c* (colored green) is then inserted to cap the cutoff components to form Ac/c*B fragments, and the caps are fused to form a molecular species c*c (concap). The caps not only perform the task to close the open valency of the components from the cut, they also represent the chemical environment of the part being cut away. Hydrogen atoms are also added to the terminal atoms in the molecular caps to avoid dangling bonds. Now the original CNT system of AB is replaced by three new subsystems, Ac, c*B and the concap c*c.

By using the MFCC ansatz^23^, the interaction energy between the CNT and an arbitrary molecule *M* can be computed by the sum of the interaction energies between the capped fragments and the molecule *M* from which the contribution of the concap is removed, as expressed by


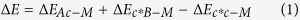


where 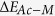 and 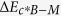 are the interaction energy of *M* with Ac and c*B molecular fragments and 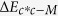
is the interaction energy of *M* with the concap c*c.

**Figure 2:**
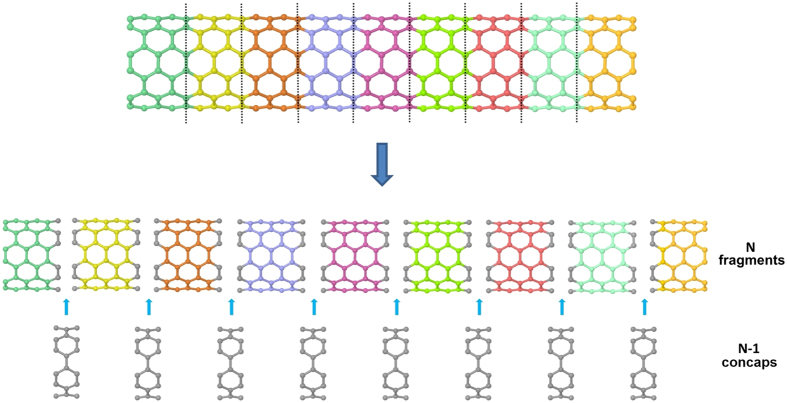
The MFCC scheme of long CNT in which a long CNT is cut into *N* fragments and *N-1* concap species. Each cut follows the MFCC scheme in Fig. 1.

The above procedure can be easily extended into larger CNT systems, which can be decomposed into multi fragments. The fragmentation procedure of long CNT is shown in Fig. 2. By partitioning the long CNT into *N* fragments and *N-1* concaps, the interaction energy of the CNT system with an arbitrary molecule *M* can be calculated by the formula


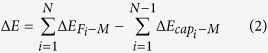


where 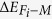 and 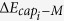 denote the interaction energy between the *i*th fragment and M, and the *i*th concap and M respectively. Using Eq. (2), the interaction energy can be obtained by simple summation over individual interaction energies between the molecule *M* and the capped CNT fragments that can be determined by *ab-initio* calculations such as HF, DFT or even higher level QM method. Obviously, the above method scales linearly with the size of the CNT and therefore applicable to large CNT interaction systems.

**Figure 3:**
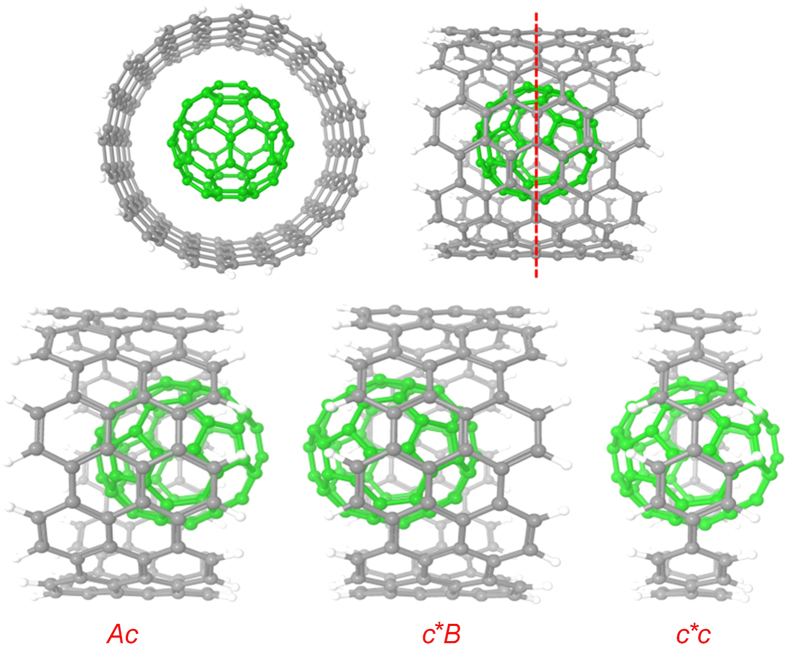
The MFCC scheme of CNT with a fullerene inside.

Then we extend our MFCC method to study hybrid nanomaterials. Encapsulated C60@CNT is a novel hybrid carbon material, which traps fullerene inside the CNT with interesting molecular properties. In order to study encapsulated C60@CNT system, we applied decomposition procedure as shown in Fig. 3. The CNT is decomposed as shown by red vertical dotted lines, and it would produce Ac, c*B fragments and c*c concap. Using the MFCC calculations on these formed fragments, the interaction energy can be determined by the summation of the contributions of all capped fragments with the contributions of all the conjugated caps being removed, which is given by Eq. (1).

### Fullerene-ligand system

**Figure 4:**
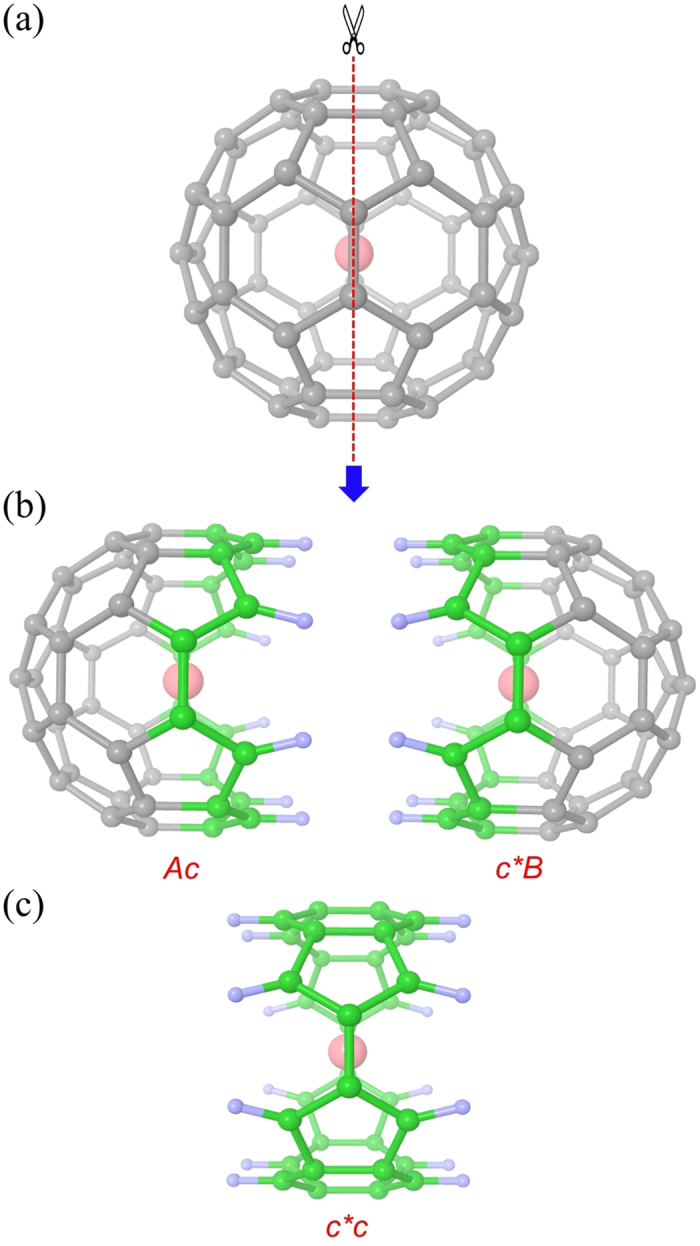
The MFCC scheme of C60 fullerene in which (**a**) a fullerene splits in two along the red dashed line, (**b**) a pair of concap (color green) is inserted to cap the hemisphere fragments and (**c**) the concap is fused to form molecular concap species.

Next, we design series of calculations to investigate the interactions of sphere type nanomaterial using C60 fullerene. C60 is similar in chemical structure to CNT, except they have closed ends to form a hollow spherical structure, which is called as buckyball. Unlike the cylindrical CNT, C60 has a limited and confined chemical environment inside its wall. They are able to hold host atoms such as water, metal ions or single molecules in their interior cages to form endohedral fullerenes. Many research groups have extensively studied the host-guest interactions of endohedral fullerenes in the past^39^,^40^,^41^,^42^,^43^,^44^,^45^,^46^,^47^,^48^,^49^,^50^. In this study, an arbitrary molecule such as water or metal cation is placed inside C60 fullerene and MFCC approach is applied to study fullerene-ligand interactions. The decomposition procedure of C60 and the formed Ac, c*B fragments and the concap c*c are shown in Fig. 4. Although C60 is a relatively small system for fragmentation, this procedure can be easily extended into large fullerenes such as C70, C84, C240 or even larger fullerenes. The interaction energy between C60 and the molecule *M* inside can be computed by separate calculations of individual fragments interacting with *M*, as given by the Eq. (1).

### Graphene-ligand system

**Figure 5:**
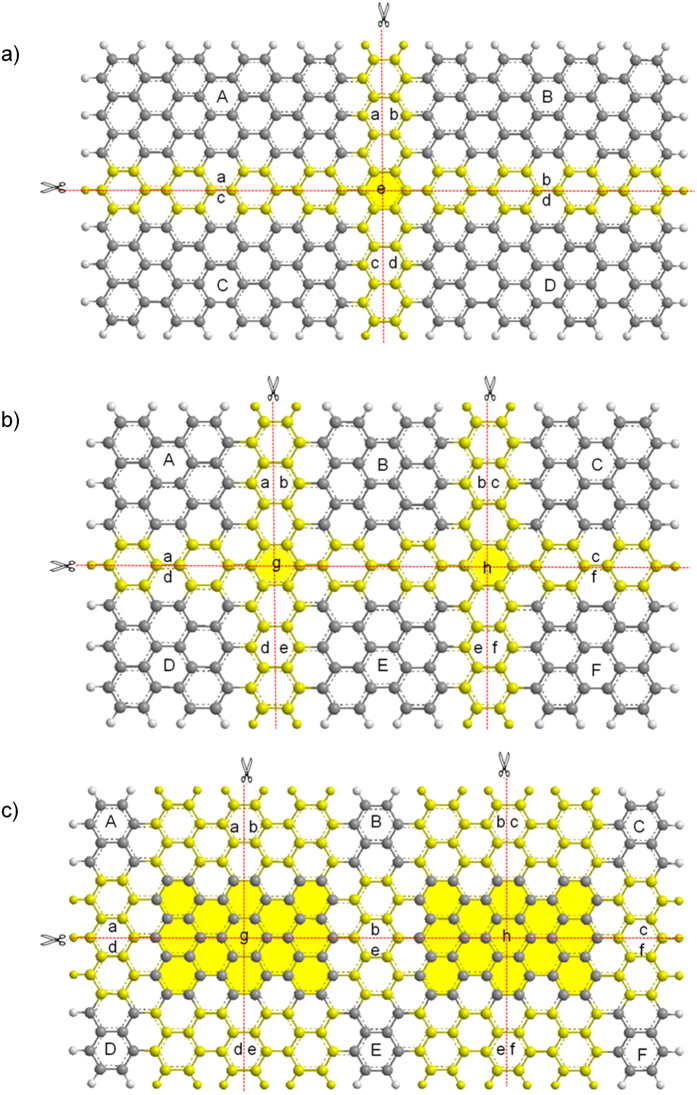
The MFCC scheme of graphene in which (**a**) a graphene is partitioned along the red dashed line to form (**a**) 4 fragments with 1-layer concap, (**b**) 6 fragments with 1-layer concap, and (**c**) 6 fragments with 3-layer concap.

Finally, we extend our MFCC study to investigate interactions of surface type carbon nanomaterials. Graphene is a planar form of sp2-bonded carbon material, which emerged as a promising nanoelectronic device along with CNTs and fullerenes. However, QM calculations of graphene sheets with large number of atoms are difficult to perform owing to the huge computational cost. MFCC method can be used as an alternative to carry out the QM studies on graphene-ligand interactions by decomposing graphene into smaller fragments. In present study, three different fragmentation methods are applied to arrange graphene fragments as shown in Fig. 5. (a) with 4 fragments, (b) with 6 fragments and 1 layer conjugated cap, and (c) with 6 fragments and 3 layer conjugated cap.

First, the graphene surface is decomposed into 4 fragments (A + B + C + D) as shown in Fig. 5a in which the interaction energy can be given by following formula.


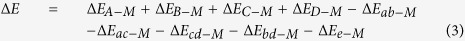


where 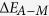, 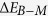,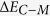, 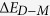 are the interaction energies of *M* with A, B, C, D molecular fragments and 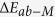, 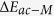, 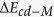, 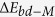, are the interaction energies of *M* with the *ab, ac, cd, bd* conjugated caps and 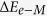 is the interaction energy of overlapped area of surface *e* (colored yellow).

Subsequently, the graphene surface is decomposed into 6 fragments (A + B + C + D + E + F) as shown in Fig. 5b, which the interaction energy can be given by following formula.


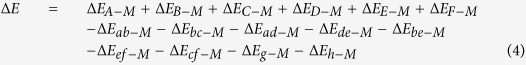


where Δ*E*_*A*−*M*_, Δ*E*_*B*−*M*_, Δ*E*_*C*−*M*_, Δ*E*_*D*−*M*_, Δ*E*_*E*−*M*_, Δ*E*_*F*−*M*_ are the interaction energies of M with A, B, C, D, E, F fragments and Δ*E*_*ab*−*M*_, Δ*E*_*bc*−*M*_, Δ*E*_*ad*−*M*_, Δ*E*_*de*−*M*_, Δ*E*_*be*−*M*_, Δ*E*_*ef*−*M*_, Δ*E*_*cf*−*M*_ are interaction energies of M with *ab, bc, ad, de, be, ef, cf* conjugated caps and Δ*E*_*g*−*M*_, Δ*E*_*h*−*M*_ are the interaction energies of M with the overlapped area of surface *g* and *h* (colored yellow).

In order to introduce different type of conjugated caps, we design series of calculations by increasing conjugated cap size from 1-layer benzene cap to 3-layer benzene cap as shown in Fig. 5c. The interaction energy between graphene and molecule *M* can be computed by separate calculations of individual fragments interacting with *M*, as same as given by the Eq. (4).

After the cut, hydrogen atoms are added in each fragment and concap using GaussView program. It should be pointed out that the geometries of the cap atoms in fragments are kept exactly the same with the geometries of the atoms in corresponding concap to ensure that the artifitial interactions between the molecule M and the concaps are cancelled. Both FS and MFCC single point energy calculations were performed with SCF convergence criterion of 10^−8^ by using the method contained ground state, DFT, default spin, B3LYP hybrid functional, with basis set 6-31G* within GAUSSIAN 09 program package^51^. During the calculation, ‘No symmetry’ keyword is used to prevent molecule reorientation so that all computations are performed in the input orientation. Since B3LYP functional lacks of the long range dispersion energy term, CNT-ligand calculations were recalculated by dispersion corrected DFT method of Grimme at B97D/6-311G* level^52^,^53^. MFCC interaction energies of each system were obtained by above given formulas and traditional single point FS computations at the same level were used as references to assess the accuracy of MFCC approach.

## Results and Discussion

### MFCC study on CNT

**Figure 6:**
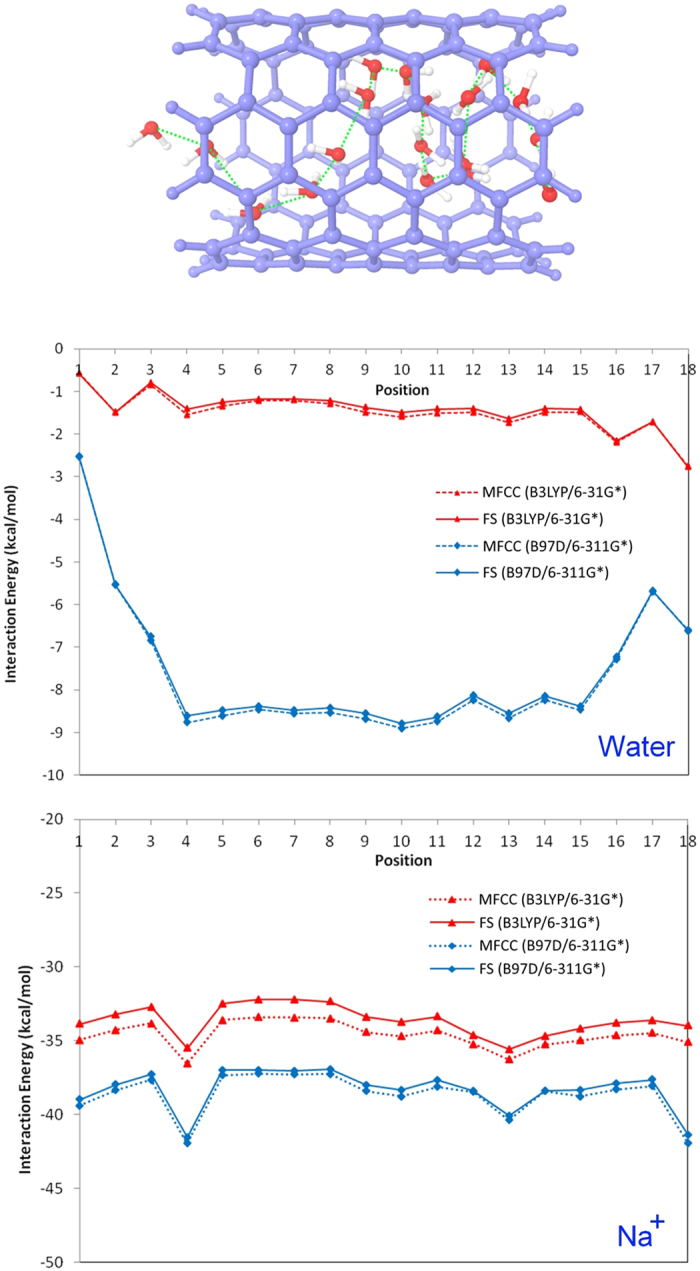
A molecule (water or Na^+^) travels inside CNT along a random pathway to generate the interaction energy curves obtained by MFCC and FS calculations using B3LYP/6-31G* and B97D/6-311G* level respectively.

As the first task in our study, MFCC method was applied to study CNT-water system. The penetration of water into the hydrophobic pore of CNT has been attracted considerable research attentions over the years, ultimately leading to the development of promising technologies for drug delivery, selective ion transport, and nano-filtration methods^54^,^55^,^56^. The behavior of water inside CNT can provide important insight into the properties of nano-confined interfacial water on hydrophobic surface. In this study, we used MFCC approach to investigate the interaction between water and (6, 6) CNT channel wall, one of the smallest diameter CNTs known to be water permeable. The water molecule was placed inside the CNT and moved inside along a random pathway as shown in Fig. 6. In the beginning, we calculated MFCC and FS interaction energies by B3LYP/6-31G* computational level. Since the dispersion energy is an important part of noncovalent interactions for CNT-water system, calculations were repeated by dispersion corrected DFT using B97D/6-311G*. As shown in Fig. 6, the MFCC and FS calculated interaction energy curves by both computational levels show good agreement with each other. The mean variation of MFCC and FS energies by B3LYP/6-31G* was calculated as 1.38 kcal/mol while the mean variation by B97D/6-311G* as 0.08 kcal/mol, showing that MFCC approach gives a good performance on both computational levels. As listed in Table 1, the average interaction energy calculated by dispersion corrected DFT is −8.0 kcal/mol, which is closer to the previously calculated energy of water molecule adsorbed in (6, 6) CNT (−8.89 kcal/mol) using two phase thermodynamics (2PT) method^57^. Evidently, B3LYP functional underestimates the CNT-water interaction, while interaction energies can be improved significantly with the dispersion corrected DFT due to the inclusion of van der Waals interaction between CNT wall and water.

The following task in our study is to examine the reliability of MFCC method in the calculation of interaction energies of CNT with charged ligand. Single charged ions marching through the CNT channel have been observed by Lee *et al*. in 2010^58^. They found that charged molecules such as sodium or chloride ions can flow rapidly through CNT and such channels can be useful as sensitive detectors or water desalination systems. In our study, MFCC approach was used to investigate the interaction of Na^+^ with CNT channel by moving Na^+^ cation inside the CNT channel. Table 2 and Fig. 6 reports the interaction energies and energy curves calculated by B3LYP/6-31G* and B97D/6-311G* computational levels. The mean deviation by B3LYP/6-31G* was calculated as 0.96 kcal/mol, while dispersion corrected DFT gave the mean deviation of 0.35 kcal/mol. Although CNT-water system shows larger interaction energy difference between two computational levels, CNT-Na^+^ system does not show such a trend, showing that motion of cationic Na^+^ inside CNT is obviously dominated by electrostatic interactions between CNT wall and charged metal cation, rather than van der Waals interactions.

**Figure 7:**
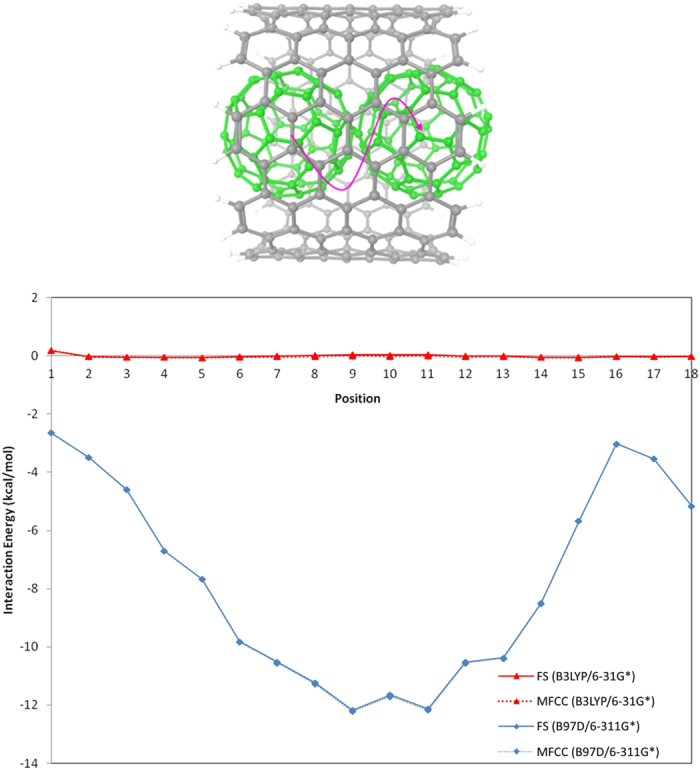
A C60 molecule travels inside CNT along a random pathway to generate the interaction energy curves obtained by MFCC and FS calculations using B3LYP/6-31G* and B97D/6-311G* level respectively.

For further extension of our study, MFCC calculations were performed on hybrid nanomaterial system of C60@CNT. C60 trapped inside CNT has been experimentally detected by Smith *et al*.^59^,^60^, showing that stable and closed carbon shells exist inside single-walled CNTs. Measurements of the diameter of these carbon shells suggested that many of them are C60 fullerenes. After that, various research groups have studied the C60@CNT systems^61^,^62^,^63^,^64^. The energetics of encapsulation of C60 inside CNT and electronic structures has been studied by Okada *et al*.^65^ and found out encapsulating process is exothermic for (10, 10) CNT, whereas the processes are endothermic for both (8, 8) and (9, 9) nanotubes. In our study, we selected a part of (15, 15) armchair CNT with radius of 10.1 Å and length of 9.8 Å with C60 molecule inside CNT and moving along a random pathway shown in Fig. 7 to generate interaction energy curves. The MFCC fragmentation procedure for CNT is exactly the same as previously mentioned as shown in Fig. 3. At first, calculations were performed at B3LYP/6-31G* computational level and then calculations were repeated at B97D/6-311G* level to include the dispersion contribution. As shown in Table 3, the B3LYP/6-31G* calculation shows the CNT-C60 interactions are almost zero. Although a change of sign between the energies calculated via FS and MFCC is observed at several positions, absolute energy value differences are still in good control to be less than 0.05 kcal/mol. When employing dispersion corrected DFT, interaction energy minimum for all the considered geometries is clearly visible. The energetics of encapsulation of C60 inside CNT has been previously studied by A. Rochefort using MM3 molecular mechanics force field and found out the reaction is exothermic with interaction energy of 12.00 kcal/mol^66^, which is similar to B97D/6-311G* calculated interaction energy of our most stable C60@CNT structure (−12.10 kcal/mol). Obviously, the B3LYP/6-31 G* calculated energies are once again underestimated significantly, while dispersion corrected DFT gives much reliable energies. The calculated interaction energies by MFCC and FS calculations using two computational levels show less than 0.01 kcal/mol deviations, showing that MFCC method is a good choice to perform QM studies on tube type nanomaterials.

### MFCC study on C60 Fullerene

**Figure 8:**
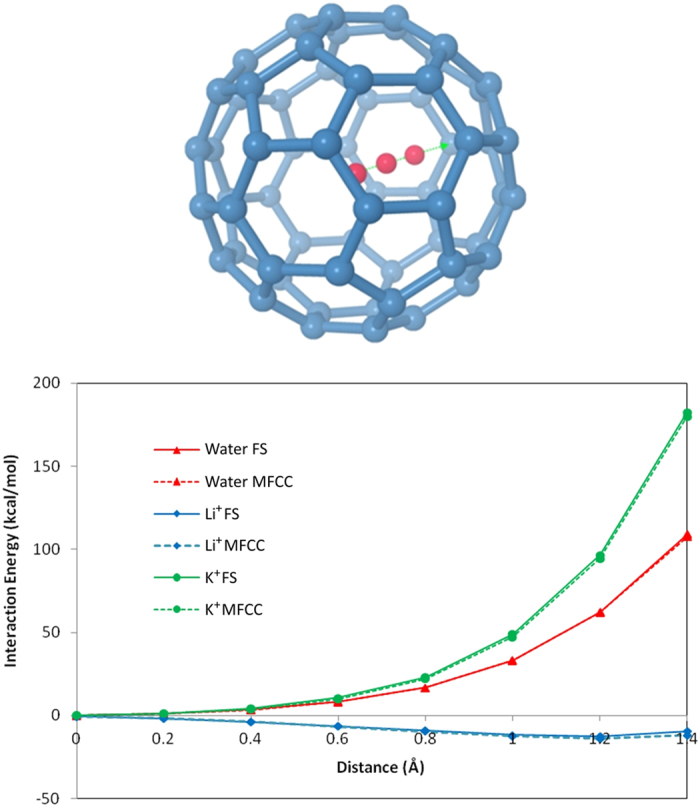
From the central position of C60, a molecule (water, Li^+^ or K^+^) moves towards the center of a 6-member ring on the C60 to generate the interaction energy curves obtained by MFCC and FS calculations using B3LYP/6-31G* and B97D/6-311G* level respectively.

Since our major aim is to explore the applicability of MFCC approach for different types of carbon nanomaterials, next we extend our study to investigate interactions of sphere type nanomaterials. The finding of endohedral fullerenes which encapsulate single molecule or metal ion inside fullerene cage is one of the most exciting finding in nanomaterial chemistry^67^,^68^. Doping various elements into C60 fullerene gave rise to the materials with novel properties and developed an entirely new branch of chemistry. Recently, Kurotabi *et al*. found that single water molecule can be confined in fullerene and intrinsic properties of water molecule, as well as physical properties of fullerene cage can be controlled by this method^69^. Here we applied MFCC approach to study the interactions of water, Li^+^ and K^+^ with C60 fullerene. The ligands were kept at the center of C60 and moved inside along one of the coordinate axes. The interaction energy variation as a function of the distance for water, Li^+^, K^+^ ligands by B3LYP/6-31G* computational level is shown in Fig. 8, while the calculated energies are summarized in Table 4. The zero position in the figure refers to the central position of the buckyball. The curves in Fig. 8 represent the one-dimensional potential energy profile when the small molecule is approaching the central position of a 6-member ring on the wall of fullerene from the central position of fullerene. As can be seen, both water and K^+^ are most stable in the center of C60 and interaction energy gradually increases with closer to the wall of C60, while Li^+^ is most stable off the center, approximately 1.2 Å away from the center of C60. It is obvious that the relative interaction energy curves computed by MFCC are in good agreement with the standard FS results with a mean deviation of 0.21, 1.00, 1.27 kcal/mol for C60-water, C60-Li^+^, and C60-K^+^ respectively. Large energy differences (above 2 kcal/mol) often come from when small molecule is very close to the inside wall of fullerene, leading to high repulsion energy. The MFCC calculation can be improved by introducing a larger cap to incorporate many-body effects more or less. The reasonable consistency of MFCC calculated energies with FS calculated values proves that MFCC method can be successfully applied to study QM interactions of sphere type nanomaterial-ligand systems.

### MFCC study on Graphene

**Figure 9:**
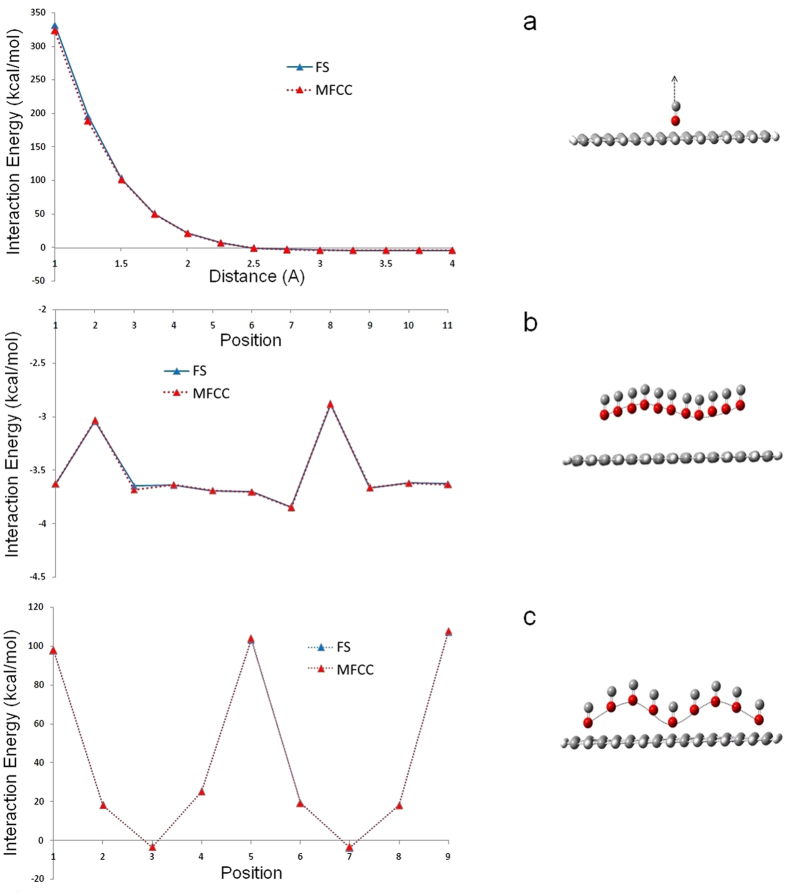
A CO molecule moves on graphene surface as shown to generate the interaction energy curves obtained by MFCC and FS calculations using B3LYP/6-31G* level.

As an effort to extend the MFCC study into larger nanomaterial systems, calculations were performed to calculate graphene-ligand interaction energies. Graphene sheets can be considered as an unrolled CNT with a monolayer of carbon atoms and similar stable physical properties to CNT. Due to its extremely large surface area and high porosity, graphene is ideal for adsorption of gases such as H_2_, CH_4_ and CO. The interaction between graphene and CO has been studied extensively due to its application in graphene-based gas sensors. In this study, we constructed a large graphene surface with CO as the ligand and MFCC calculations were performed by decomposing the surface into smaller fragments. Since the most stable adsorption site for CO on pristine graphene has been found to be at hollow site^70^, we started our study by keeping CO 1.00 Å away from the hollow site of one benzene ring. In order to generate interaction energy curves, CO molecule was gradually moved away from the surface. All the calculated energies are summarized in Table 5, which shows the CO-graphene interaction is highly repulsive when CO is closer to the surface and gradually stabilizes when moving away from the surface. As shown in the Fig. 9a, the calculated graphene-CO interaction energies by both FS and MFCC methods show a good agreement with only 0.13 kcal/mol mean deviation for energy values below 10 kcal/mol, confirming the reliability of MFCC approach.

Next, we continued the MFCC study by decomposing the large graphene sheet into smaller fragments to check the effect of fragment size on calculated interaction energies. To this aim, we decomposed the graphene surface into 6 fragments as shown in Fig. 5b and extended our study by moving CO ligand vertically with slight oscillation on the graphene surface. The interaction energies calculated by MFCC and FS are plotted in Fig. 9b and listed in Table 6. As can be seen, interaction energy values depend on the graphene-CO distance and ranges from −2.8 to −3.8 kcal/mol. It is obvious that our MFCC method well reproduced the FS interaction energies with mean deviation only a fractional kcal/mol, which proves the reliability of MFCC approach for studying large graphene surfaces using smaller fragments. Although the MFCC fragmentation of graphene surface into a number of smaller fragments is a little complicated, it decreases the computational cost of each fragment and allows faster evaluation of MFCC interaction energies.

In order to verify the effect of the size of conjugated caps on the calculated interaction energies, additional series of calculations were designed by increasing the conjugated cap size into 3 benzene layers as shown in Fig. 5c. The graphene surface was decomposed into 6 fragments and CO ligand moved diagonally on the surface with a higher oscillation movement. As can be seen in Table 7 and Fig. 9c, the interaction energies are fluctuating between highly positive and negative values, depending on the graphene-CO distance. The largest deviation of graphene-CO interaction energies between FS and MFCC calculations is less than 0.5 kcal/mol, which suggests that accuracy of MFCC is not affected by the size of conjugated caps. Since larger cap makes MFCC computation more expensive because their capping enlarges the size of the fragments and increases the computational cost, employing a smaller cap as possible is enough to get accurate MFCC interaction energies. Obviously, all the three MFCC fragmentation procedures used on graphene surface give satisfactory results, indicating our fragmentation and capping procedure provides a new alternative approach for QM interaction energy calculations of surface type nanomaterials.

## Conclusion

We have performed a series of MFCC benchmark calculations to study nanomaterial-ligand interactions by decomposing three types of nanomaterials, namely carbon nanotube, fullerene, and graphene into small fragments respectively. Unlike the application of MFCC approach on large biomolecules which is more complicated and difficult to assess the percentage error with FS results, its application on carbon nanomaterials can be an ideal benchmark system to evaluate the accuracy of MFCC approach due to simplicity and orderness of nanomaterial structure. Obviously, our benchmark tests demonstrate that MFCC on various forms of nanomaterials gives consistently accurate interaction energies with very small deviations compared to the corresponding FS calculated energies. Even so, the MFCC scheme still brings some errors. Considering the interaction energy curves (Figs 6, 7, 8, 9), we can see roughly that the MFCC scheme gives smaller or more negative interaction energies compared with the FS calculations. The part of the missing values is probably due to not fully considering the many-body effects. When the FS calculation produces a very small positive energy value, the MFCC scheme is likely to give a negative energy value, resulting in a change of sign between the energies calculated via FS and MFCC.

To illustrate the computational expense of our MFCC approach, information about average calculation time of FS and MFCC calculations is given in Table 8. As an example, the computational time for a single point FS calculation on graphene surface is about two days, while less than 6 hours for MFCC fragment calculations. And the advantage on computing time becomes more prominent with higher correlation methods and for larger nanomaterial systems due to the linear scaling feature of the MFCC method versus standard N^3^ for HF/DFT method and N^5^ for MP2 method. An additional attractive feature of the MFCC method is that its *ab initio* calculation can be easily parallelized to run on multi-node computer clusters that could dramatically speed up the computation.

The excellent agreement of MFCC calculated energies with FS values indicates that MFCC method is a promising theoretical tool for fast and reliable calculation of *ab-initio* interaction energies of nanomaterials with both neutral and charged ligands. This method is almost linear scaling with system size and thus makes it possible to obtain interaction energies and molecular properties even for large nanomaterial-ligand systems.

## Additional Information

**How to cite this article:** Zhang, D. Quantum mechanical calculation of nanomaterial-ligand interaction energies by molecular fractionation with conjugated caps method. *Sci. Rep.* **7**, 44645; doi: 10.1038/srep44645 (2017).

**Publisher's note:** Springer Nature remains neutral with regard to jurisdictional claims in published maps and institutional affiliations.

## Figures and Tables

**Table 1:** Interaction energies of CNT-water system (Energies are in kcal/mol).

Position	B3LYP/6-31G*				B97D/6-311G*			
	FS	MFCC	Absolute difference†	Percentage absolute difference (%)‡	FS	MFCC	Absolute difference†	Percentage absolute difference (%)‡
1	−0.582	−0.577	0.005	0.86	−2.535	−2.534	0.001	0.04
2	−1.494	−1.486	0.008	0.54	−5.539	−5.537	0.002	0.04
3	−0.795	−0.843	0.048	6.04	−6.754	−6.834	0.080	1.18
4	−1.410	−1.545	**0.135**	9.57	−8.603	−8.762	**0.159**	1.85
5	−1.244	−1.339	0.095	7.64	−8.471	−8.599	0.128	1.51
6	−1.181	−1.216	0.035	2.96	−8.379	−8.465	0.086	1.03
7	−1.180	−1.208	0.028	2.37	−8.475	−8.551	0.076	0.90
8	−1.213	−1.285	0.072	5.94	−8.422	−8.530	0.108	1.28
9	−1.375	−1.486	0.111	8.07	−8.553	−8.679	0.126	1.47
10	−1.497	−1.594	0.097	6.48	−8.789	−8.902	0.113	1.29
11	−1.412	−1.509	0.097	6.87	−8.633	−8.744	0.111	1.29
12	−1.401	−1.489	0.088	6.28	−8.130	−8.231	0.101	1.24
13	−1.638	−1.729	0.091	5.56	−8.557	−8.667	0.110	1.29
14	−1.407	−1.491	0.084	5.97	−8.140	−8.236	0.096	1.18
15	−1.418	−1.485	0.067	4.72	−8.390	−8.468	0.078	0.93
16	−2.157	−2.187	0.030	1.39	−7.230	−7.278	0.048	0.66
17	−1.714	−1.718	0.004	0.23	−5.676	−5.698	0.022	0.39
18	−2.775	−2.755	0.020	0.72	−6.612	−6.598	0.014	0.21

The maximum absolute difference between the two approaches is represented with bold text.

^†^Absolute difference = |MFCC − FS|.

^‡^Percentage absolute difference (%) = |Absolute difference/FS|.

**Table 2:** Interaction energies of CNT-Na^+^ system (Energies are in kcal/mol).

Position	B3LYP/6-31G*				B97D/6-311G*			
	FS	MFCC	Absolute difference†	Percentage absolute difference (%)‡	FS	MFCC	Absolute difference†	Percentage absolute difference (%)‡
1	−33.852	−34.925	1.073	3.17	−38.966	−39.364	0.398	1.02
2	−33.184	−34.267	1.083	3.26	−37.957	−38.344	0.387	1.02
3	−32.713	−33.806	1.093	3.34	−37.259	−37.638	0.379	1.02
4	−35.486	−36.539	1.053	2.97	−41.523	−41.939	0.416	1.00
5	−32.478	−33.590	1.112	3.42	−36.978	−37.340	0.362	0.98
6	−32.191	−33.387	**1.196**	3.72	−36.976	−37.216	0.240	0.65
7	−32.220	−33.416	1.196	3.71	−37.022	−37.261	0.239	0.65
8	−32.333	−33.479	1.146	3.54	−36.922	−37.240	0.318	0.86
9	−33.374	−34.393	1.019	3.05	−37.969	−38.394	0.425	1.12
10	−33.694	−34.678	0.984	2.92	−38.316	−38.754	0.438	1.14
11	−33.345	−34.303	0.958	2.87	−37.656	−38.100	0.444	1.18
12	−34.611	−35.217	0.606	1.75	−38.364	−38.442	0.078	0.20
13	−35.589	−36.243	0.654	1.84	−40.048	−40.360	0.312	0.78
14	−34.668	−35.253	0.585	1.69	−38.388	−38.376	0.012	0.03
15	−34.150	−34.964	0.814	2.38	−38.340	−38.765	0.425	1.11
16	−33.787	−34.623	0.836	2.47	−37.857	−38.290	0.433	1.14
17	−33.614	−34.460	0.846	2.52	−37.621	−38.058	0.437	1.16
18	−33.965	−35.066	1.101	3.24	−41.364	−41.895	**0.531**	1.28

The maximum absolute difference between the two approaches is represented with bold text.

^†^Absolute difference = |MFCC − FS|.

^‡^Percentage absolute difference (%) = |Absolute difference/FS|.

**Table 3:** Interaction energies of C60@CNT system (Energies are in kcal/mol).

Position	B3LYP/6-31G*				B97D/6-311G*			
	FS	MFCC	Absolute difference†	Percentage absolute difference (%)‡	FS	MFCC	Absolute difference†	Percentage absolute difference (%)‡
1	0.181	0.181	0.000	—	−2.645	−2.646	0.001	0.04
2	−0.034	−0.034	0.000	—	−3.498	−3.499	0.001	0.03
3	−0.050	−0.048	0.002	—	−4.593	−4.592	0.001	0.02
4	−0.057	−0.055	0.002	—	−6.705	−6.705	0.000	0.00
5	−0.058	−0.061	0.003	—	−7.668	−7.672	0.004	0.05
6	−0.021	−0.039	0.018	—	−9.819	−9.839	0.020	0.20
7	0.002	−0.031	0.033	—	−10.518	−10.554	0.036	0.34
8	0.022	−0.021	0.043	—	−11.239	−11.287	0.048	0.43
9	0.048	−0.003	**0.051**	—	−12.170	−12.224	0.054	0.44
10	0.033	−0.018	0.051	—	−11.647	−11.702	**0.055**	0.47
11	0.044	−0.001	0.045	—	−12.128	−12.177	0.049	0.40
12	−0.001	−0.032	0.031	—	−10.521	−10.555	0.034	0.32
13	−0.007	−0.027	0.020	—	−10.373	−10.395	0.022	0.21
14	−0.049	−0.056	0.007	—	−8.500	−8.508	0.008	0.09
15	−0.059	−0.056	0.003	—	−5.672	−5.670	0.002	0.04
16	−0.026	−0.026	0.000	—	−3.022	−3.023	0.001	0.03
17	−0.031	−0.031	0.000	—	−3.542	−3.544	0.002	0.06
18	−0.018	−0.020	0.002	—	−5.164	−5.166	0.002	0.04

The maximum absolute difference between the two approaches is represented with bold text. The percentage absolute differences for the calculations based on B3LYP/6-31G* are neglected since a change of sign between the energies calculated via FS and MFCC is observed at several positions, leading to abnormally large percentage error.

^†^Absolute difference = |MFCC − FS|.

^‡^Percentage absolute difference (%) = |Absolute difference/FS|.

**Table 4:** Interaction energies of C60-water/Li^+^/K^+^ systems (Energies are in kcal/mol).

Distance (Å)	FS	MFCC	Absolute difference†	Percentage absolute difference (%)‡
C60-water				
0.00	0.099	0.070	0.029	29.3
0.20	1.076	1.033	0.043	4.00
0.40	3.478	3.418	0.060	1.73
0.60	8.254	8.218	0.036	0.44
0.80	16.855	16.849	0.006	0.04
1.00	33.141	33.217	0.076	0.23
1.20	62.034	62.162	0.128	0.21
1.40	109.189	107.503	**1.686**	1.54
C60-Li^+^				
0.00	−0.508	−0.537	0.029	5.71
0.20	−1.778	−1.847	0.069	3.88
0.40	−3.745	−3.847	0.102	2.72
0.60	−6.259	−6.512	0.253	4.04
0.80	−9.068	−9.558	0.490	5.40
1.00	−11.665	−12.560	0.895	7.67
1.20	−12.594	−14.062	1.468	11.7
1.40	−9.346	−11.674	**2.328**	24.9
C60-K^+^				
0.00	0.297	0.206	0.091	30.6
0.20	1.393	1.181	0.212	15.2
0.40	4.212	3.797	0.415	9.85
0.60	10.586	9.819	0.767	7.25
0.80	23.289	22.099	1.190	5.11
1.00	49.014	47.328	1.686	3.44
1.20	96.658	94.525	2.133	2.21
1.40	182.662	180.149	**2.513**	1.38

The maximum absolute difference between the two approaches is represented with bold text.

^†^Absolute difference = |MFCC − FS|.

^‡^Percentage absolute difference (%) = |Absolute difference/FS|.

**Table 5:** Interaction energies of graphene-CO/4 fragment system (Energies are in kcal/mol).

Distance (Å)	FS	MFCC	Absolute difference†	Percentage absolute difference (%)‡
1.00	331.164	323.578	**7.586**	2.34
1.25	195.703	189.384	6.319	3.34
1.50	103.307	101.672	1.635	1.61
1.75	50.540	49.762	0.778	1.56
2.00	21.908	21.596	0.312	1.44
2.25	7.580	7.435	0.145	1.95
2.50	−0.800	−0.076	0.724	—
2.75	−2.143	−3.014	0.871	28.9
3.00	−3.386	−3.387	0.001	0.03
3.25	−3.768	−3.769	0.001	0.03
3.50	−3.843	−3.846	0.003	0.08
3.75	−3.806	−3.811	0.005	0.13
4.00	−3.747	−3.753	0.006	0.16

The maximum absolute difference between the two approaches is represented with bold text. The percentage absolute differences for the distance of 2.50 Å is neglected since the FS energy is almost zero, leading to abnormally large percentage error.

^†^Absolute difference = |MFCC − FS|.

^‡^Percentage absolute difference (%) = |Absolute difference/FS|.

**Table 6:** Interaction energies of graphene-CO/6 fragment system (Energies are in kcal/mol).

Position	FS	MFCC	Absolute difference†	Percentage absolute difference (%)‡
1	−3.626	−3.624	0.002	0.06
2	−3.042	−3.029	0.013	0.43
3	−3.647	−3.679	**0.032**	0.88
4	−3.638	−3.633	0.005	0.14
5	−3.690	−3.688	0.002	0.05
6	−3.701	−3.701	0.000	0.00
7	−3.841	−3.848	0.007	0.18
8	−2.883	−2.882	0.001	0.03
9	−3.662	−3.663	0.001	0.03
10	−3.620	−3.619	0.001	0.03
11	−3.625	−3.633	0.008	0.22

The maximum absolute difference between the two approaches is represented with bold text.

^†^Absolute difference = |MFCC − FS|.

^‡^Percentage absolute difference (%) = |Absolute difference/FS|.

**Table 7:** Interaction energies of graphene-CO/6 fragment system with 3-benzene layer conjugated cap (Energies are in kcal/mol).

Position	FS	MFCC	Absolute difference†	Percentage absolute difference (%)‡
1	97.458	97.817	0.359	0.37
2	18.131	18.106	0.025	0.14
3	−3.444	−3.431	0.013	0.38
4	25.367	25.014	0.353	1.39
5	103.140	103.930	**0.790**	0.77
6	19.497	19.136	0.361	1.85
7	−3.974	−3.434	0.540	13.6
8	18.295	18.063	0.232	1.27
9	107.173	107.757	0.584	0.54

^†^Absolute difference = |MFCC − FS|.

^‡^Percentage absolute difference (%) = |Absolute difference/FS|.

The maximum absolute difference between the two approaches is represented with bold text.

**Table 8:** Average computational time of FS and MFCC fragments (B3LYP/6-31G*).

Nanomaterial	QM method	No of Atoms	Time (min)
CNT	FS	132	247
	MFCC	96	105
C60	FS	60	96
	MFCC	54	69
C60@CNT	FS	390	1252
	MFCC	240	496
Graphene	FS	320	2741
	MFCC	118	341
